# The paradox of prosperity and poverty: confronting inequality in Norway

**DOI:** 10.1016/j.lanepe.2025.101225

**Published:** 2025-02-03

**Authors:** The Lancet Regional Health – Europe

Norway is celebrated worldwide for its wealth, exceptional living standards, and robust welfare system. With a gross domestic product per capita far surpassing the Organisation for Economic Co-operation and Development (OECD) average and a consistent spot as one of the top ten countries in the World Happiness Report, Norway epitomises prosperity and wellbeing. Yet, despite its characteristic egalitarianism, a widening socioeconomic gradient threatens to deepen health inequalities. Notably, although Norway ranked first in the World Happiness Report in 2017, its position has since steadily declined, settling at seventh since 2023—a trend that warrants reflection on the factors shaping national wellbeing.

The proportion of individuals living in low-income households in Norway has gradually increased from 8.1% to 10.1% over the past decade, with poverty disproportionately affecting immigrant populations. In 2022, over 30% of individuals born outside of Norway and the European Union were at risk of poverty or social exclusion, compared with just below 15% for those born in Norway. These figures underscore the urgency of addressing the socioeconomic factors contributing to poverty and inequality in Norway.

Alongside the gradual increase in overall poverty, alarmingly, child poverty in Norway increased by 10.1% between 2012–14 and 2019–21. As highlighted by Věra Skalická and Terje Andreas Eikemo in their Comment, childhood poverty has substantial effects on physical and mental health and can affect socioeconomic attainment in later life. Therefore, public health policies should aim to tackle poverty in childhood to avoid perpetuating inequalities in health through generations.

A 2024 report on health and wellbeing inequalities in Norway, published by the Institute of Health Equity, predicts that these income inequalities will continue to drive poor health outcomes, particularly among vulnerable groups, such as single-parent households, immigrants, and individuals with low educational attainment. To address poverty in Norway seriously, key policy interventions need to be taken now to strengthen income redistribution mechanisms, improve access to affordable housing, expand educational and employment opportunities for individuals with low socioeconomic status, and ensure a robust safety net for those most at risk of falling behind.

Key policy interventions should focus on strengthening income redistribution through progressive taxation and enhanced social transfers (eg, cash benefits, pensions, and subsidies for essential services), expanding affordable housing initiatives, and regulating rental markets. Investments in job creation, upskilling programmes, and improved access to early childhood education can boost opportunities for individuals with low socioeconomic status. Targeted support for vulnerable populations, alongside measures to address health inequalities and ensure food security, are essential. Subsidised healthy food options and free school meal programmes can further mitigate the impact of poverty. Robust monitoring, transparent reporting, and policy evaluation are crucial to assess the effectiveness of these measures and ensure sustained progress. Lastly, the national government must hold municipalities accountable for implementing these interventions and prioritising health equity.

Although the current centre-left minority coalition Norwegian Government has poverty and health and social inequalities high on their agenda, a stronger approach is needed to reduce inequalities effectively and must be maintained in the face of potential leadership changes. The next parliamentary elections are set for Sept 8, 2025, and polls suggest a potential shift in Norway's leadership, with the right-wing Progress Party leading at 26%. If they secure power, their focus on health-care competition, privatisation, and tax exemptions for the wealthy could deprioritise poverty reduction. Privatised hospitals could target healthier, profitable patients, leaving those with multimorbidities—often from lower-income groups—more dependent on an overburdened public system, exacerbating health inequalities. This approach risks worsening care quality and outcomes, further marginalising vulnerable populations.

Given that Norway contributes the highest proportion of its national income to international aid among OECD countries—far exceeding the UN target of 0.7%, which many nations fail to meet—it is paradoxical that such stark inequalities persist domestically. While Norway's global leadership in aid is commendable, the growing internal divide demands urgent attention. The wealth of the top 10%—largely concentrated within the top 0.1%—has surged, while the bottom 50% have seen little change. This disparity poses both a moral concern and a risk to long-term economic stability. The Norwegian Government must address this paradox, finding ways to foster equity at home while maintaining its global philanthropic leadership. Action is needed now to ensure that Norway's prosperity benefits all, and leaves nobody behind.

